# Study of changes in neuropsychological indicators in adults

**DOI:** 10.1192/j.eurpsy.2021.1912

**Published:** 2021-08-13

**Authors:** A. Sidenkova

**Affiliations:** Psychiatry, Ural State Medical University, Yekaterinburg, Russian Federation

**Keywords:** Pathological brain aging, cognitive functions during aging

## Abstract

**Introduction:**

Currently, the number of cases of pathological aging of the CNS, represented by a violation of cognitive functions, is increasing. But there is a social request to prolong the physical and mental activity of older people

**Objectives:**

The study of the dynamics of cognitive aging is timely and relevant. The article contains a report on a cohore non-repeating study of higher brain functions at various age periods

**Methods:**

The average age was 45.1 + 5.7 years. Inclusion criteria: 1. Dextral. Non-inclusion criteria: 1. Clinically significant somatic diseases in their medical history. 2. Mental disorders in their medical history. •Applied neuropsychological, statistical research methods. The research tool was the neuropsychological rapid method including the subtests: •“Memorizing 9 words in three presentations (1st, 2nd, 3rd attempts)”, •“Sequential subtraction”, •“Test of Benton’s visual memory”, •“Solving an arithmetic problem”, •“Overlaid images”, •“Specified flow of associations in 1 minute”, • “Figure of 3 geometric figures”, • “Blind hours”, • “Graph-motor test”, “Delay word reproduction”

**Results:**

The first cohort 27–40 years old. The second cohort 41–50 years old.Third cohort 51 years old and older. A significant difference in the performance of the graphomotor test between the subjects of the age subgroup of 27-40 years and the subgroup of 41-50 years was statistically confirmed. In older people revealed a much greater number of errors, interruptions of the test than the representatives of the more “young” subgroup

**Conclusions:**

The deterioration in the performance of the graphomotor test was the most age-specific

**Disclosure:**

No significant relationships.

